# Characterization Analysis of *Schistosoma japonicum* Plasma Membrane Repair Relative Gene Myoferlin

**DOI:** 10.1371/journal.pone.0066396

**Published:** 2013-06-18

**Authors:** Yanian Xiong, Ming Zhang, Yang Hong, Meimei Wei, Dezhou Ai, Peipei Meng, Yanhui Han, Zhiqiang Fu, Yaojun Shi, Jianmei Yang, Jiaojiao Lin

**Affiliations:** National Laboratory of Animal Schistosomiasis Control/Key Laboratory of Animal Parasitology, Ministry of Agriculture, Shanghai Veterinary Research Institute, Chinese Academy of Agricultural Sciences, Shanghai, People’s Republic of China; Queensland Institute of Medical Research, Australia

## Abstract

Myoferlin is a member of the ferlin family of proteins, which are involved in plasma membrane repair, and has been identified as one of the tegument proteins of *Schistosoma japonicum.* The tegument proteins are potential candidates for vaccines and new drug targets. In this study, myoferlin of *S. japonicum* (SjMF) was cloned, expressed and characterized, the potential of SjMF recombinant protein (rSjMF) as a vaccine candidate was evaluated, and the effect of praziquantel on SjMF was detected by Real-time PCR. Immunofluorescence showed that this protein was mainly distributed on the surface of worms at different stages. Sequence analysis revealed that the SjMF open reading frame was conserved at all stages of the *S. japonicum* life cycle. And SjMF transcription was upregulated in 42-day-old worms, and was significantly higher in female worms. Western blotting revealed that rSjMF showed strong immunogenicity. The cytokine profile and IgG isotype analysis demonstrated that rSjMF plus ISA206 immunization induced a mixed T helper (Th)1/Th2 response. Purified rSjMF emulsified with ISA206 adjuvant significantly reduced worm burden from 21.8% to 23.21% and liver egg number from42.58% to 28.35%. Besides, SjMF transcription was downregulated when worms were exposed to low-dose praziquantel (PZQ) and upregulated when PZQ was degraded, accompanied by recovery of damaged tegument. When worms were exposed to high-dose PZQ, SjMF transcription was downregulated all the time and the damaged tegument did not recover. These findings indicated that SjMF is a potential vaccine against *S. japonicum* and provides the basis for further investigations into the biological function of SjMF.

## Introduction

Schistosomes are parasitic blood helminths that infect millions of people in tropical and subtropical countries [1]. Approximately 779 million people are at risk of being infected in 76 endemic countries and an estimated 280,000 deaths are directly or indirectly attributable to the disease annually [2], [3]. Besides humans, >40 types of livestock and wild animals are reservoir hosts for *Schistosoma japonicum* in China, and cattle are the major source of infection, especially in the lake–marsh endemic area of the Yangtze River. Therefore, schistosomiasis control remains a major challenge in China. Currently, schistosomiasis control strategy is mainly based on treatment of infected individuals with praziquantel (PZQ). PZQ can effectively reduce the morbidity associated with schistosomiasis, but it has been proved not to be sufficient to control disease transmission and prevent reinfection [4], [5]. An effective vaccine against schistosomiasis would be essential to the current control strategy, mainly because it would provide long-lasting immunity against infection. In addition, it is suggested that the combined use of chemotherapy and vaccination is the basis for a novel, more versatile method to control schistosomiasis. Therefore, it is important to identify the appropriate schistosomal antigens that could induce activity against schistosomal infection or reduce the release of live eggs to limit parasite transmission.

The ability of schistosomes to survive in the inhospitable environment of the mammalian bloodstream and avoid host immune responses can be attributed in part to their tegument [6]. Schistosomal proteins on the surface of the tegument that are exposed to the host may be ideal molecules for the discovery of vaccine candidates and drug targets. Indeed, some surface proteins such as Tetraspanins of Schistosoma mansoni (SmTSP) [7] and others [8]–[10] have proved to be high-efficacy vaccine candidates against schistosomal infection. Based on proteomics study of tegument surface proteins of *S. japonicum* in our laboratory, myoferlin, which belongs to the ferlin family, was identified as one of the tegument proteins of this parasite. Previous studies have demonstrated that ferlin family members containing dysferlin, myoferlin, and otoferlin, play a role in calcium-mediated membrane fusion events [11]. Based on their involvement in vesicular fusion, the ferlin proteins are supposed to be candidates for mediating membrane repair. A recent study has found that the levels of myoferlin mRNA and protein are downregulated in healthy myofibers and upregulated in response to myofiber damage [12]. Davis et al. Have found that myoferlin is expressed abundantly in both cardiac and skeletal muscle and is associated with the plasma and nuclear membranes [11]. Doherty et al. have suggested that the interaction of myoferlin with eps15 homology domain protein (EHD2) may facilitate membrane fusion at sites of contact between cells where cytoskeletal rearrangements are needed [13]. Furthermore, Robinson et al. have validated the expression of myoferlin in term placenta and trophoblastic cells, and have speculated that myoferlin also repairs damage to the syncytiotrophoblast apical plasma membrane [14].

In the present study, we described the cloning, expression, and immunolocalization of the myoferlin of *S. japonicum* (SjMF) gene, as well as the immunogenicity of recombinant SjMF (rSjMF). We also evaluated the protective immunity induced by rSjMF, and the effect of PZQ on SjMF transcription.

## Materials and Methods

### Ethics Statement

All animal care and procedures were conducted according to the guidelines for animal use in toxicology (Society of Toxicology USP, 1989). The study protocol was approved by the Animal Care and Use Committee of the Shanghai Veterinary Research Institute, Chinese Academy of Agricultural Sciences.

### 1. Mice and Parasites

Male BALB/c mice aged 6 weeks were purchased from Shanghai Experimental Animal Centre, Chinese Academy of Sciences. The life cycle of *S. japonicum* was maintained routinely in *Oncomelania hupensis* snails and New Zealand rabbits, and cercariae were obtained by exposing infected snails to light for shedding. The number of cercariae and their viability were determined using a light microscope. Parasites at 7, 13, 21, 28, 35, and 42 days were obtained by perfusion of artificially infected New Zealand rabbits. Partial 42-day-old worms were manually separated into males and females.

### 2. Molecular Characterization of SjMF

The deduced amino acid sequence of SjMF was analyzed as follows: theoretical isoelectric point (pI) and molecular weight (MW) were calculated at http://www.expasy.org/tools/pi tool.html; prediction of signal peptides with SignalP 3.0 at http://www.cbs.dtu.dk/services/SignalP; search for glycosylphosphatidyl anchors in the sequence with NetNGlyc 1.0 at http://www.cbs.dtu.dk/services/NetNGlyc/; analysis of conserved domains using ScanProsite at http://www. ebi.ac.uk/Tools/pfa/iprscan/; prediction of transmembrane helices using the TMHMM Server v.2.0 at http://www.cbs.dtu.dk/services/TMHMM-2.0; and protein modeling at http://swissmodel.expasy.org/. The amino acid sequences of the myoferlin protein were obtained from GenBank and were aligned using ClustalX software (http://www.clustal.org/).

### 3. Cloning and Sequence Analysis of the ORF of SjMF c-DNA

The forward and reverse oligonucleotides, 5′- ATAGGATCCAT GGTAGATTCACAG TGGG-3′ and 5′-ATACTCGAGGGCATCAGTATAGGCAGG-3′ (*Bam*HI and *Xho*I sites are underlined), were used to amplify the complete SjMF open reading frame (ORF; GenBank accession AAW27277.1) from a transcriptome cDNA library of worms at different developmental stages by PCR (the polymerase chain reaction) with Phusion High-Fidelity PCR Master Mix. Amplification was performed in an initial denaturation step at 98°C for 1 min, then 32 cycles at 98°C for 8 s, 52°C for 1 min, and 72°C for 90 s, and a post-PCR step at 72°C for 10 min. dATP was added to the blunt end of the purified PCR product by DNA A-Tailing Kit (TaKaRa). Then, the PCR-generated fragment was cloned into the pMD19-T vector (TaKaRa) and sequenced. To understand the structural conservation of SjMF in worms at different developmental stages, c-DNA encoding the ORF of SjMF was cloned by PCR with Phusion High-Fidelity PCR Master Mix. Then, the PCR-generated fragment was cloned into the pMD19-T vector (TaKaRa) and 40 clones of each stage were sequenced and compared.

### 4. Real-time Reverse-transcriptase PCR (RT-PCR) Analysis of SjMF

Transcription of SjMF at the mRNA level was evaluated in 7-, 14-, 21-, 28-, 35-, and 42-day-old worms of *S. japonicum*, as well as in 42-day-old female and male worms using real-time quantitative RT-PCR. Total RNA was isolated from the different developmental stages of worms using TRIzol reagent (Invitrogen) according to the manufacturer’s instructions. RNA samples were treated with RNase-free DNaseI (Takara) and purified with an RNeasy Mini Kit (QIAGEN, USA) following the manufacturer instructions. RNA was quantified by spectrophotometry (Biophotometer; Eppendorf, Germany). cDNA was synthesized using PrimeScript RT Reagent Kit with gDNA Eraser (Takara) according to standard protocols. A product of 222 bp was amplified with the primers designed for SjMF (forward primer: 5′-GCACAGTTGGCGATTTCT-3′; reverse primer: 5′-GGCTTCTCGGTAGGCTTT-3′). A negative control which did not include cDNA as a template was set in each PCR run. Real-time PCR was performed in a reaction mixture of 20 µl containing 10 µl 2× SYBR Green PCR Premix Taq (TaKaRa), 6.8 µl EASY Dilution Buffer (TaKaRa), 2 µl cDNA, 0.8 µl primers (10 µM), and 0.4 µl ROX Reference Dye II (50×). The cycling protocol was 95°C for 30 s followed by 40 cycles of 95°C for 5 s, 60°C for 34 s, and a dissociation stage of 95°C for 15 s, 60°C for 1 min, and 95°C for 15 s in a ABI PRISM 7500 Fast Real-Time PCR System. The generation of a specific PCR product was tested using melting curve analysis and sequenced. And the efficiency of the PCR reaction was between 95% and 105%. The independent experiments were repeated three times. Differences in transcription levels were observed by comparison to the housekeeping gene, NADH dehydrogenase with double-standard curves method of relative quantification PCR [15], [16].

### 5. Transcription Level Analysis of SjMF by Treatment with PZQ

Ninety mice were divided randomly into nine groups of 10 Mice were challenged through percutaneous exposure of abdominal skin for 15 min in water containing 100 viable cercaria. Thirty-five days after challenge, the infected groups were treated with a single dose of PZQ at 40 or 200 mg/kg in carboxyl methyl cellulose (CMCNa). Infected mice were sacrificed at 30 min, 4 h, 12 h, and 36 h after drug administration. The effect of PZQ on SjMF transcription between the treated and untreated groups was determined by RT-PCR. Scanning electron microscopy (SEM) and transmission electron microscopy (TEM) were used to examine the ultrastructural differences in the tegumental and subtegumental structures between the worms treated with PZQ at different doses. After treatment with PZQ, worms were collected and fixed in 4% formaldehyde (in 0.1 M cacodylate buffer, pH 7.4) overnight at 4°C, and processed according to a standard method [17]. Worms were rinsed three times with ice-cold cacodylate buffer (0.1 M, pH 7.4) and post-fixed in buffered osmium tetroxide, followed by dehydration with decreasing concentrations of alcohol before critical point drying. The specimens were mounted on stubs and sputter-coated with gold, and examined under a JEOL-6380LV scanning electron microscope. For TEM, after fixation, post-fixation and dehydration, worms were embedded in spur resin. Ultrathin sections of the schistosomes were stained with uranyl acetate and lead citrate. The specimens were then examined under a Tecnai G2 Spirit BioTwin transmission electron microscope.

### 6. Expression and Purification of Recombinant Protein

With overhanging ends generating from *Bam*HI and *Xho*I, the cDNA of SjMF was subcloned into the multiple cloning sites present in the pET32a(+) expression vector (Invitrogen) to produce a fusion protein. The recombinant plasmid pET32a(+)-SjMF was transformed into *Escherichia coli* BL21(DE3) (Tiangen Biotech Co. Ltd., Beijing, China), and transformants were confirmed by restriction enzyme digestion and sequence analysis. Transformed *E. coli* BL21(DE3) cells were grown in 500 ml Luria–Bertani medium plus ampicillin (100 µg/ml) at 37°C with shacking until OD_600_ reached 0.6, and overexpressed using 1 mM isopropyl-β-D- thiogalactopyranoside (IPTG) at 37°C for 6 h. The bacteria were harvested by centrifugation at 10,000×g for 15 min. Following this, the pellet was suspended in 15 ml phosphate buffered saline (PBS, pH 7.4) and extracted using an ultrasonic processor to release the fusion proteins. The lysates were centrifuged at 12,000×g for 15 min to collect inclusion bodies and cellular debris, while leaving other soluble substances. The pellets were resuspended in 5 ml 1× binding buffer plus 6 M urea. After analyzed by SDS-PAGE, rSjMF was expressed mainly in an inclusion body form. rSjMF was purified using a Ni-NTA His-Bind Resin (Qiagen GmbH, Hilden, Germany) and dialyzed against PBS (pH 7.4), containing decreasing concentrations of urea (6, 4, 3, 2, and 1 M) and PBS only. The purified recombinant protein was used to inject BALB/c mice emulsified with ISA206 adjuvant three times to produce polyclonal antibodies, which were used for the following immunolocalization test.

### 7. Immunolocalization of SjMF

To analyze the tissue distribution of SjMF, freshly collected adult worms were embedded in optimal cutting temperature (OCT) compound medium and precooled in a freezing microtome cryostat for 30 min, then cut into 8-µm sections for immunofluorescence assay. The sections were fixed with precooled acetone for 30 min and immunolabeled using indirect immunofluorescence as follows. The sections were blocked with 10% goat serum in PBS containing 0.05% Tween 20 (PBST) for 2 h at 37°C, and incubated with the anti-rSjMF mouse serum diluted 1∶200 in blocking buffer for 1 h at 37°C. Serum from nonimmunized mice was used as a negative control. After three washes in PBST, samples were probed with CY3-conjugated goat-anti-mouse IgG (Rockland, ME, USA), diluted 1∶3,000 in blocking buffer for 1 h at 37°C. Sections were washed three times and stained with 10 µg/ml 49,6-diami-dino-2-phenylindole (DAPI) for 8 min at room temperature, and observed by fluorescence microscopy (Nikon, Japan).

### 8. Western Blot Analysis

The purified rSjMF protein was subjected to 12% SDS-PAGE and transferred electrophoretically onto a 0.45-µm pore nitrocellulose membrane (Whatman, Germany) at 130 mA for 1 h at 4°C. The membranes were blocked with PBST plus 5% skimmed milk at 37°C for 2 h, washed three times with PBST, and probed with serum from rabbits immunized against SWAP (soluble adult worms antigen preparation) diluted 1∶200 for 1 h at 37°C. After three washes in PBST, membranes were probed with goat-anti-rabbit IgG conjugated to horseradish peroxidase (HRP) diluted 1∶3000 in PBST for 1 h. Following a further three washes, the result was visualized using precipitation-type TMB Substrate Solution (Tiangen Biotech).

### 9. Evaluating the Protective Efficacy of rSjMF

Six-week-old male BALB/c mice (10 mice per group) were immunized subcutaneously with 20 µg rSjMF emulsified with ISA206 adjuvant on days 0, 15 and 30. In the control groups, adjuvant in PBS or PBS alone was administered using the same immunization protocol. On day 10 after each immunization, blood samples from 10 mice in each experimental group were collected by retro-orbital bleeding and stored at –20°C until use. Two weeks after the last boost, mice were challenged through percutaneous exposure of abdominal skin for 15 min in water containing 40±1 viable cercaria, as described by Smithers and Terry [18]. 42 days post-challenge, adult worms were perfused from the portal system and mesenteric veins. The protection level was calculated by comparing the number of worms recovered from the immunization group with that of the control group, using the formula: protection level = (worms recovered from the control group – worms recovered from the vaccination group)/worms recovered from the control group × 100%) [9].

To evaluate the liver egg burden, liver from each mouse in the control and rSjMF immunized groups were collected 42 days post-infection. One gram samples of liver tissue from each mouse were digested in 10 ml 5% NaOH for 30 min at 56°C for 30 min, and mixed thoroughly. An average of three counts per 20 µl mixture was taken to estimate the number of eggs, and this count was converted to eggs per gram (EPG). The reduction in liver EPG was calculated using the formula: protection level = (EPG from the control group – EPG from the vaccinated group)/EPG from the control group × 100%).

### 10. Measurement of Specific Antibodies

The measurement of specific anti-SjMF IgG, IgG1 and IgG2a antibodies was performed by ELISA. Maxsorp 96-well microtiter plates were coated with 100 µl/well rSjMF(10 µg/ml) in carbonate–bicarbonate buffer, pH 9.6, for 16 h at 4°C. The plates were then blocked for 2 h at 37°C with 150 µl/well PBST plus 1.5% bovine serum albumin. One hundred microliters of each serum sample, diluted 1∶200 in PBST, was added per well and incubated for 1 h at 37°C. Plate-bound antibody was detected using goat-anti-rabbit IgG conjugated to horseradish peroxidase (Sigma–Aldrich, St. Louis, MO, USA), goat-anti-rabbit IgG1 conjugated to horseradish peroxidase (AbD Serotech, Kidlington, UK), and goat-anti-rabbit IgG2a conjugated to horseradish peroxidase (AbD Serotech) diluted in PBST to 1∶3000. Color reaction was developed by the addition of 100 µl/well TMB (Tiangen Biotech) and stopped with 50 µl/well 5% sulfuric acid. Absorbance was measured at 450 nm in an ELISA reader.

### 11. Cytokine Analysis

Cytokine analysis was performed using serum from five mice 7 days after the third immunization with rSjMF emulsified with 206 adjuvant or 206 adjuvant in PBS. The level of stimulated cytokine was analyzed by the Luminex 100 multiplex bead-based immunoassay system by labeled cytokine capture antibody pairs (Bio-Plex Suspension Array Systembio-plex System; Bio-Rad, Hercules, CA, USA) according to the manufacturer’s procedures. Stimulated levels of interleukin (IL)-12p70, interferon (IFN)-γ, IL-4 and IL-10 cytokines were determined using Bio-Plex Manager Software (Bio-Rad). All standards and samples were run in triplicate as recommended.

### 12. Statistical Analysis

Statistical analyses were performed with paired and unpaired Student’s *t* test or ANOVA using the software package GraphPad Prism 4.0 (GraphPad Software, San Diego, CA, USA).

## Results

### 1. Molecular Cloning and Sequence Analysis of SjMF

**Figure 1:**
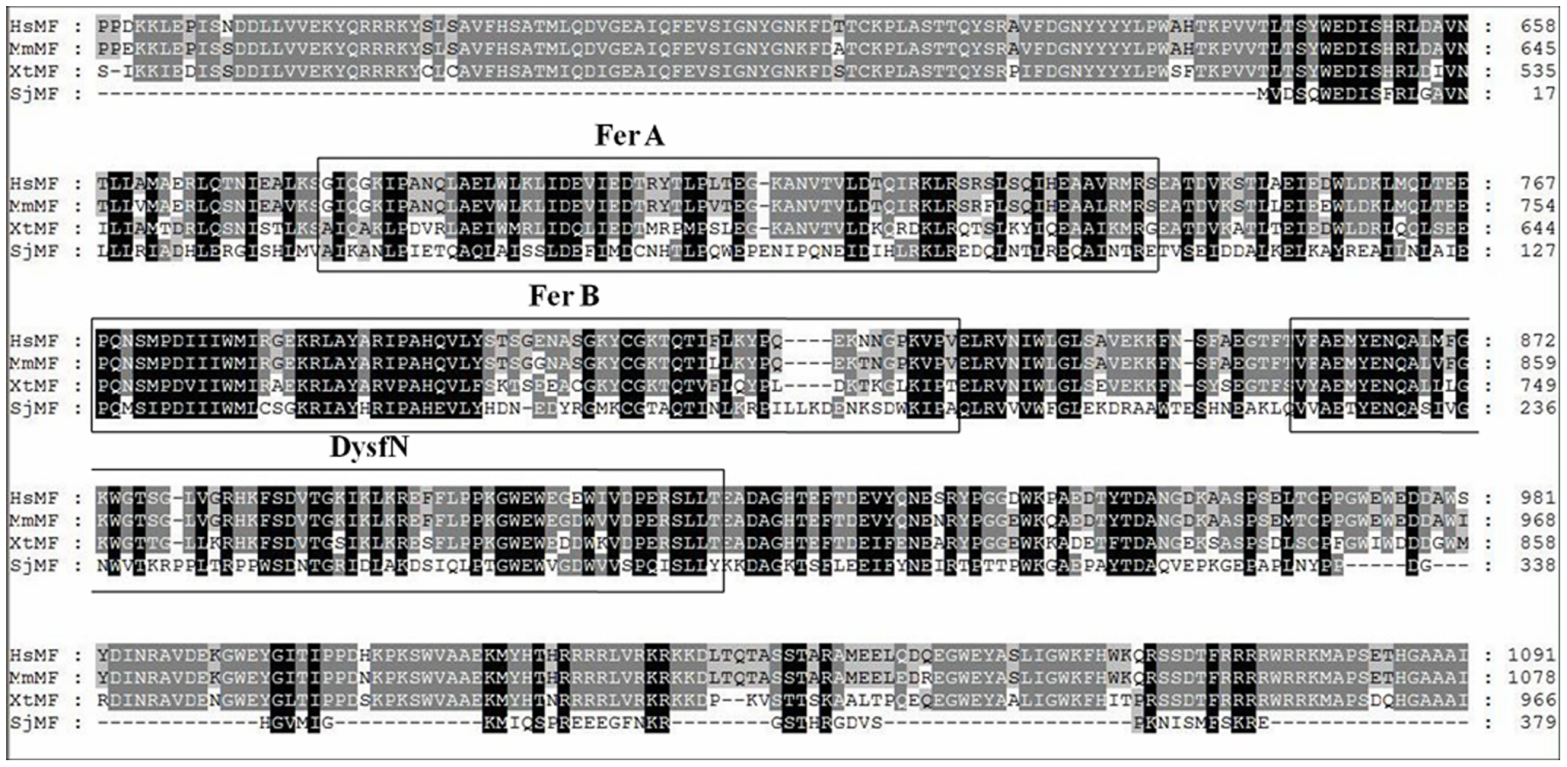
Clustal X alignment of the derived amino acid sequences of SjMF (AAW27277.1), HsMF (Q9NZM1), MmMF (Q69ZN7) and XtMF (B3DLH6). The regions with high identity and similarity among myoferlin sequences are shown as black and gray columns, according to the Clustal X algorithm. The boxes refer to FerA (central domain A in proteins of the ferlin family), FerB (central domain B in proteins of the ferlin family), and DysfN (Dysferlin domain, N-terminal region) domain respectively.

The sequence of the *S. japonicum* cDNA encoding SjMF was obtained by PCR amplification with specific oligonucleotides. The sequences of 40 cDNA clones encoding the ORF of SjMF from 7-, 14-, 21-, 28-, 35-, and 42-day-old worms were analyzed and compared. All the sequences were conserved in worms from each stage of the parasitic life cycle tested. And there were five substitutions in the deduced amino acid sequence compared with the original sequence deposited in GenBank (AAW27277.1). The coding sequence showed an ORF of 963 bp and encoded a protein of 321 amino acids with a predicted molecular mass of approximately 37,050 Da and a pI of 5.16. Post-translational modifications were assessed in silico, and the results revealed that SjMF did not contain a signal peptide, N-glycosylation sites, or transmembrane helices. After searching with BlastX, comparison of the amino acid sequences showed that the myoferlin of *S. japonicum* had 40%, 40% and 41% identity with its orthologs in *Xenopus tropicalis*, *Mus musculus* and *Homo sapiens*, respectively. The consensus sequence showed that the FerA (central domain A in proteins of the ferlin family), FerB (central domain B in proteins of the ferlin family), and DysfN (Dysferlin domain, N-terminal region) domain was conserved among all of these species (Fig. 1).

### 2. Transcription Levels of SjMF at Different Stages of the S. japonicum Life Cycle

**Figure 2:**
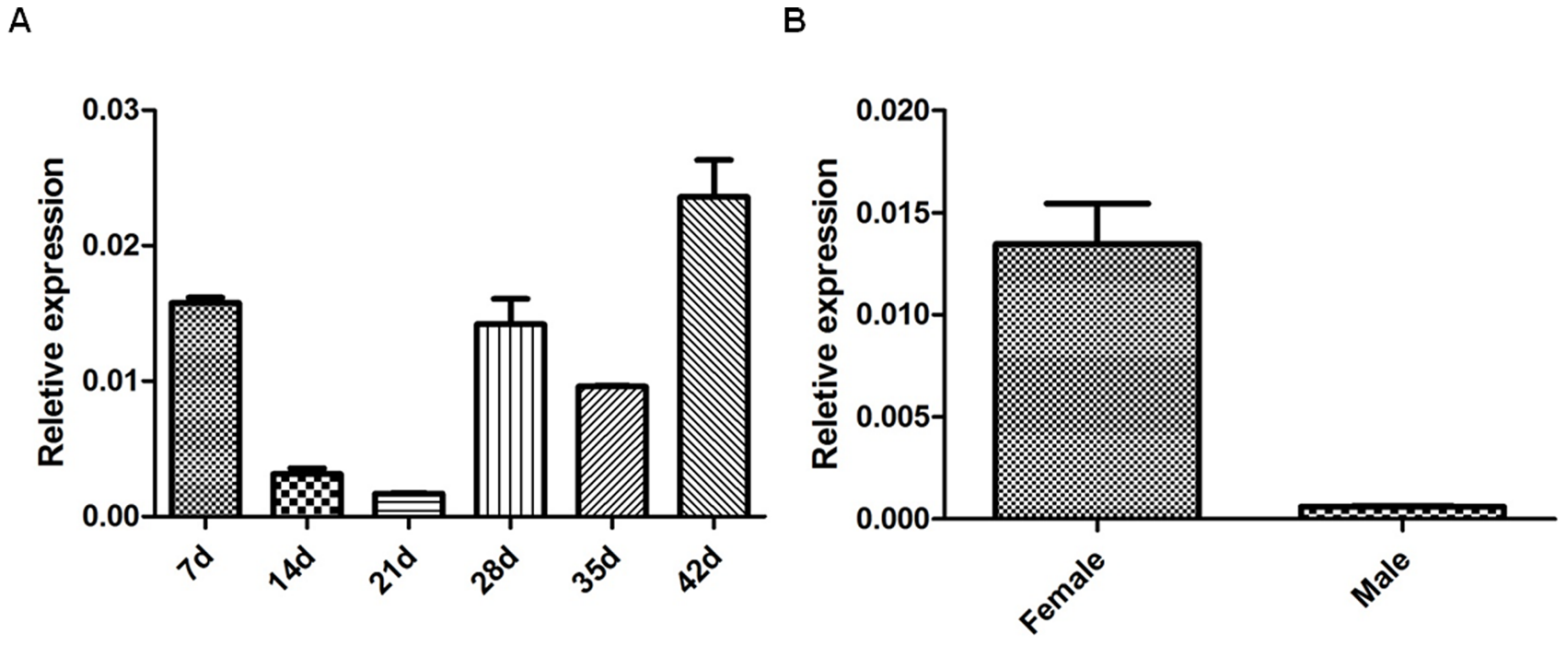
Stage and sex differential expression of SjMF in *S. japonicum*. (A) Expression throughout six stages of *S. japonicum*, and (B) expression in male and female adult worms analyzed by real-time PCR. Data were normalized against amplification of an internal housekeeping control gene SjNADH. The data are the means ± SD of one representative of three independent experiments.

The results showed that SjMF was transcripted at all developmental stages tested and exhibited a higher transcription level in 42-day-old worms and lower transcription level in 21-day and 14-day schistosomula (Fig. 2). The results also suggested that transcription level in the 42-day female worms were higher than in their male counterparts.

### 3. Dose Dependent Effect of PZQ on SjMF Transcription and Tegumental Damage

**Figure 3:**
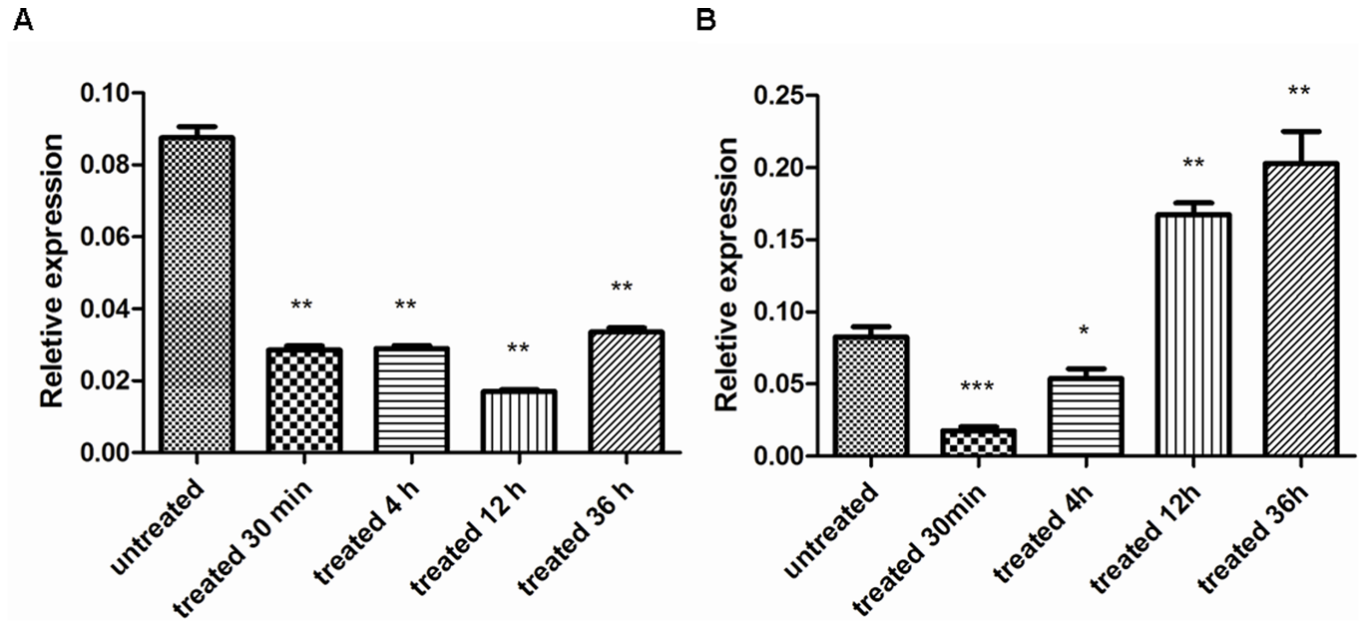
Effect of praziquantel on the SjMF transcription by real-time RT-PCR. (A) Mice treated with single dose of 200 mg/kg PZQ and sacrificed at various times post-treatment. (B) Mice treated with single dose of 40 mg/kg PZQ and sacrificed at various times post-treatment. Statistically significant compared with the control group denoted by *P<0.05, **P<0.01 or ***P<0.001.

**Figure 4:**
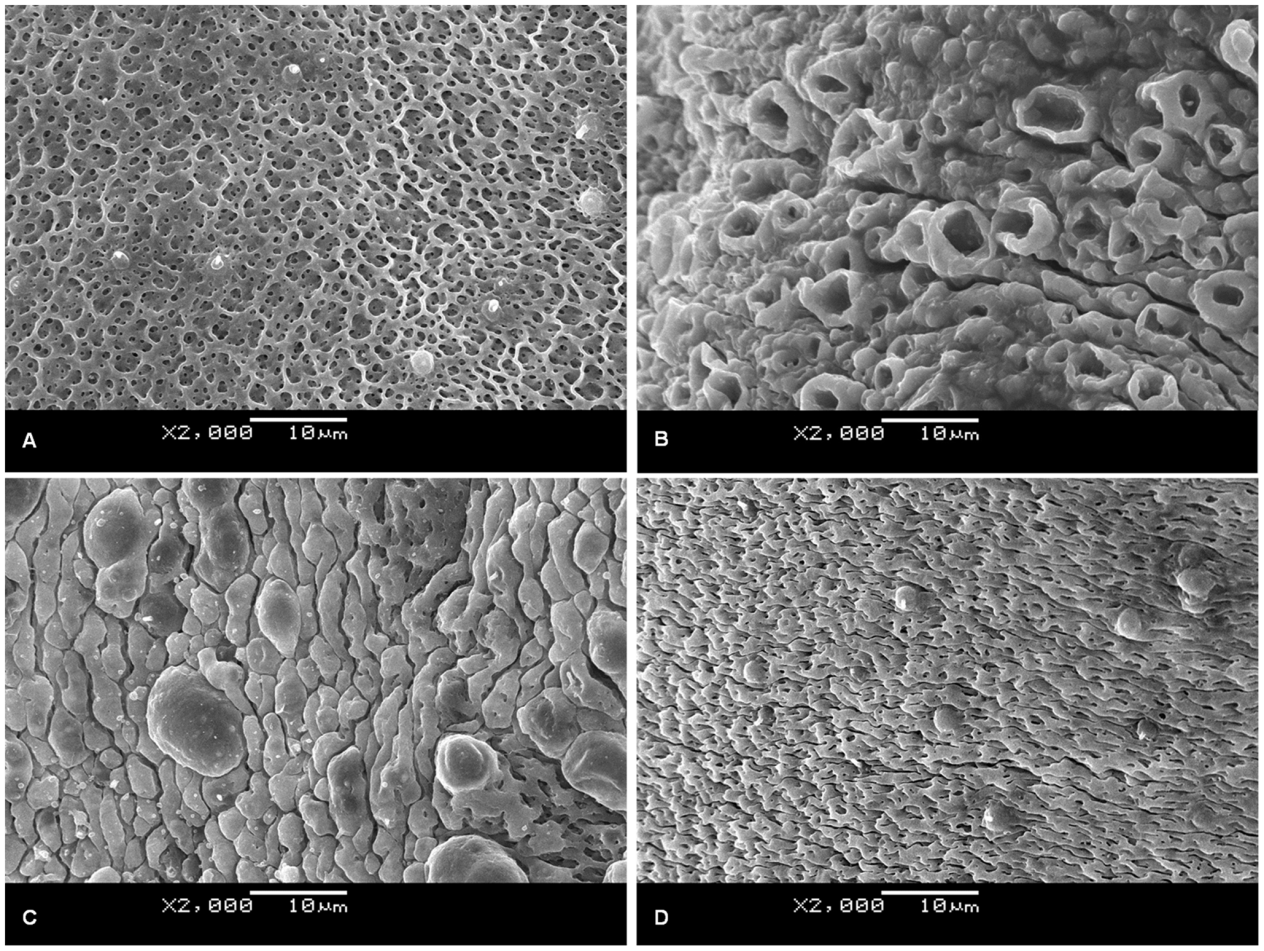
Photomicrographs of the dorsal surface of male, adult *S. japonicum* as observed by SEM. These micrographs provide a visual sample of the comparative damage induced by PZQ in the control group (A), in a mouse at 36 h after treatment with 200 mg/kg PZQ (B), a mouse at 4 h (C) or 36 h (D) after treatment with 40 mg/kg PZQ.

**Figure 5:**
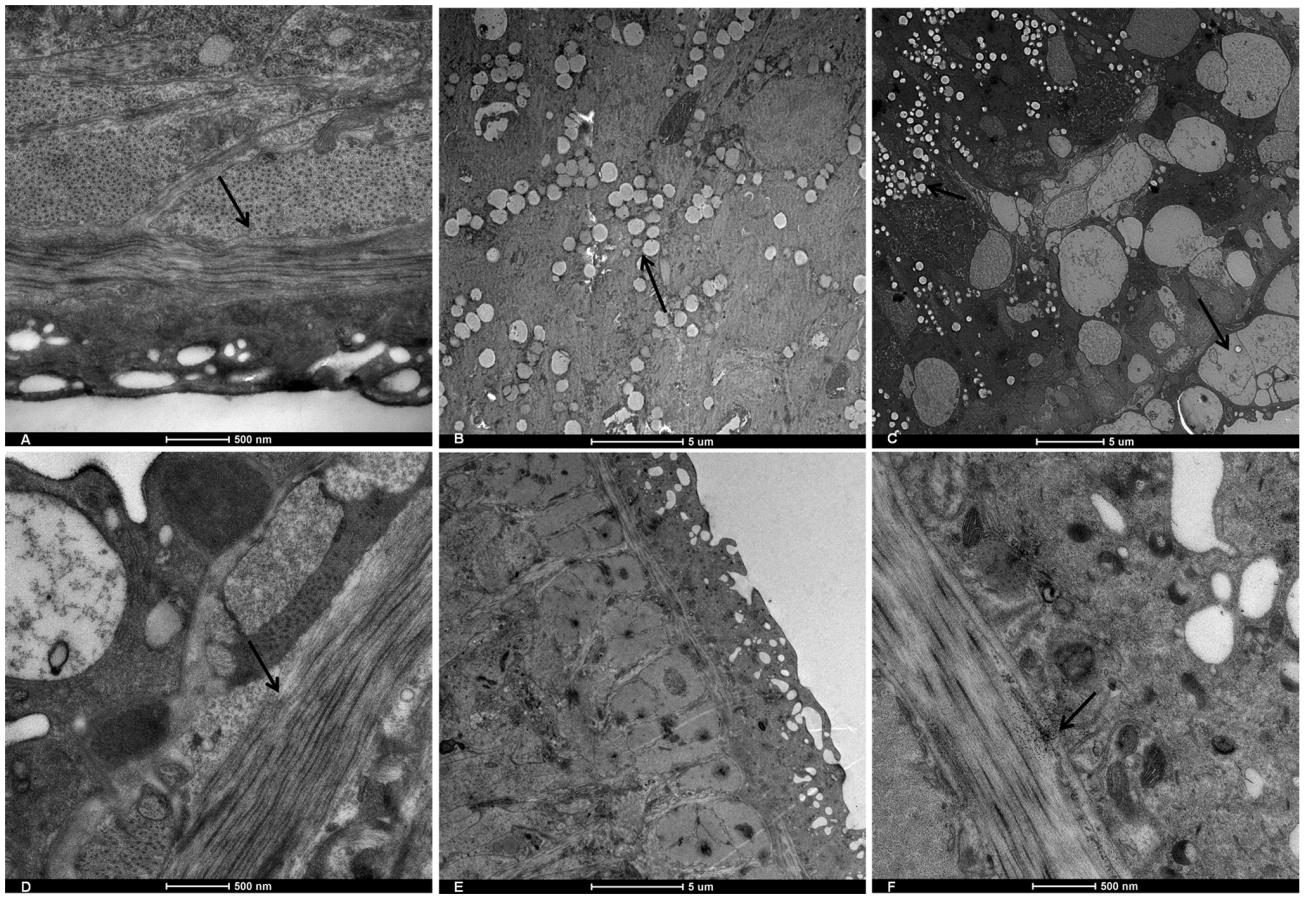
TEM observation of ultrastructural alterations in *S. japonicum* caused by PZQ. These micrographs provide a visual sample of the comparative damage induced by PZQ in the control group (A), 36 h after treatment with 200 mg/kg PZQ (B), 4 h after treatment with 40 mg/kg PZQ (C, D), and 36 h after treatment with 40 mg/kg PZQ (E, F). The outer plasma membrane was smooth and integral and musculature was regular (arrow in A) in the control group. The tegumental matrix and parenchymal tissues lost their definition and became indistinct and there was focal or extensive lysis of muscle bundles and parenchymal tissues, which resulted in vacuole formation (arrow in B). Plates C showed vacuole formation in the matrix ((large arrow in C) and in parenchymal tissues (small arrow in C). The underlying muscle bundles were swollen (small arrow in D). The surface returned to its normal morphological appearance and the large vacuoles were decreased and turned substantial (Plates E), but the muscle bundles were still swollen (arrow in F).

SjMF transcription was significantly inhibited at 36 h after treatment with a single dose of 200 mg/kg PZQ (Fig. 3A). However, when treated with a single dose of 40 mg/kg PZQ, SjMF transcription was significantly inhibited after 30 min, recovered partly after 4 h, and then upregulated significantly after 12 and 36 h (Fig. 3B). The surface of adult worms from untreated mice was composed of grooves and channels arranged regularly and the tegument was distinct and intact (Figs. 4A and 5A), which was similar to that described previously [19]. Severe damage to the tegument was observed at 36 h after treatment with 200 mg/kg PZQ. The typical structural features were almost completely degenerated, and much of the corrugated appearance was either swollen or collapsed (Fig. 4B). Meanwhile, the tegumental matrix and parenchymal tissues lost their definition and became indistinct and there was focal or extensive lysis of muscle bundles and parenchymal tissues, which resulted in vacuole formation (Fig. 5B). When treated with 40 mg/kg PZQ, after 4 h, the tegumental surface was ballooned and swollen (Fig. 4C). The tegumental matrix and underlying muscle bundles were also swollen (Fig. 5D), and there was extensive lysis and some formation of small vacuoles (Fig. 5C) in parenchymal tissues and large vacuoles (Fig. 5C) in the tegument, but the structure remained intact (Figs. 4C, 5C and 5D). The damage to the tegument was recovered partially, accompanied by upregulation of SjMF, at 36 h after treatment. The surface returned to its normal morphological appearance and the large vacuoles were decreased and turned substantial, but the muscle bundles were still swollen (Figs. 4D, 5E and 5F).

### 4. SjMF is Mainly Located in the Tegument of S. japonicum

**Figure 6:**
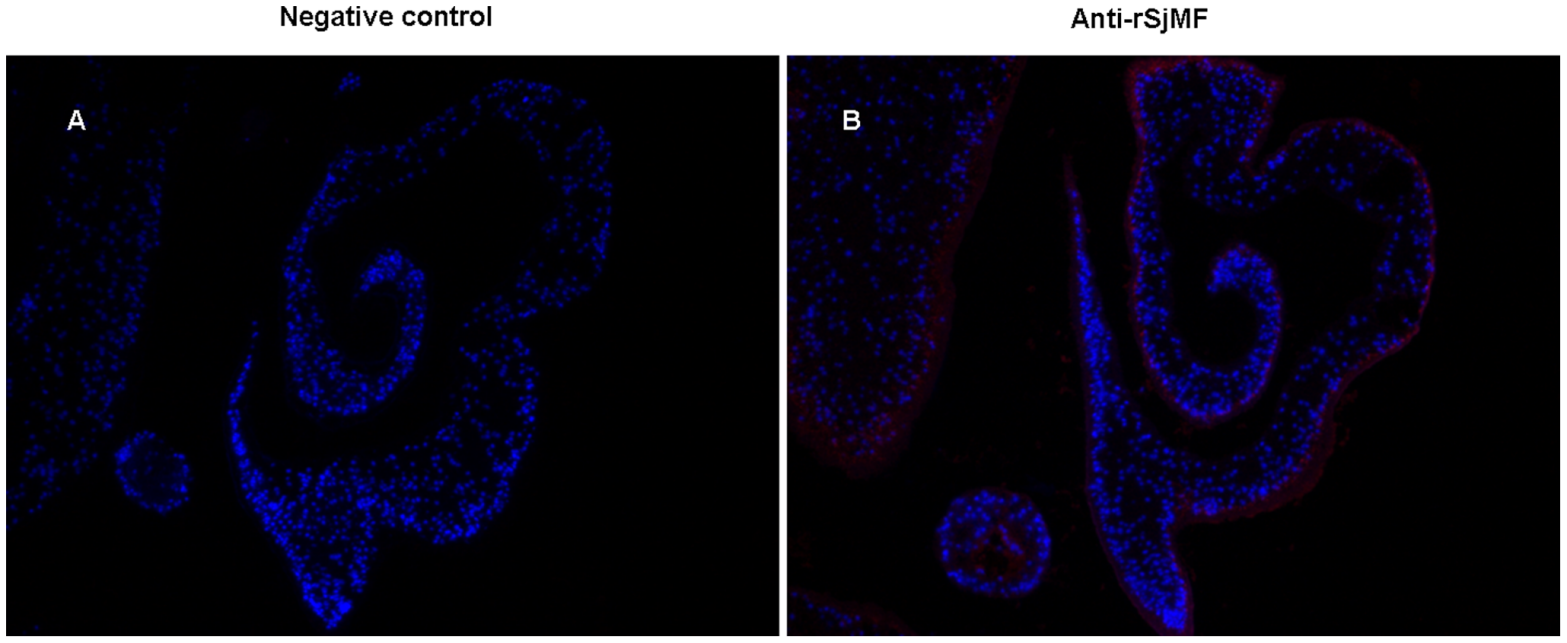
Immunolocalization of SjMF in the *S. japonicum* adult worms and lung-stage schistosomes. Secondary antibody CY3-conjugated anti-mouse IgG (red) was used for fluorescence detection of SjMF on adult worms and lung-stage schistosome sections. DAPI (blue) was used to stain parasite nuclei. (A) Section was probed with naïve mouse serum (negative control). (B) Section of *S. japonicum* adult worms probed with anti-rSjMF mouse serum.

Immunolocalization assay was performed to identify the distribution of the SjMF protein in the *S. japonicum* with anti-rSjMF and naive mouse serum. Native SjMF was mainly distributed over the tegument and at lower levels in the internal tissues of the parasite, however, no specific staining was observed in sections incubated with naive mouse serum (Fig. 6).

### 5. Immunogenicity Analysis of rSjMF

**Figure 7:**
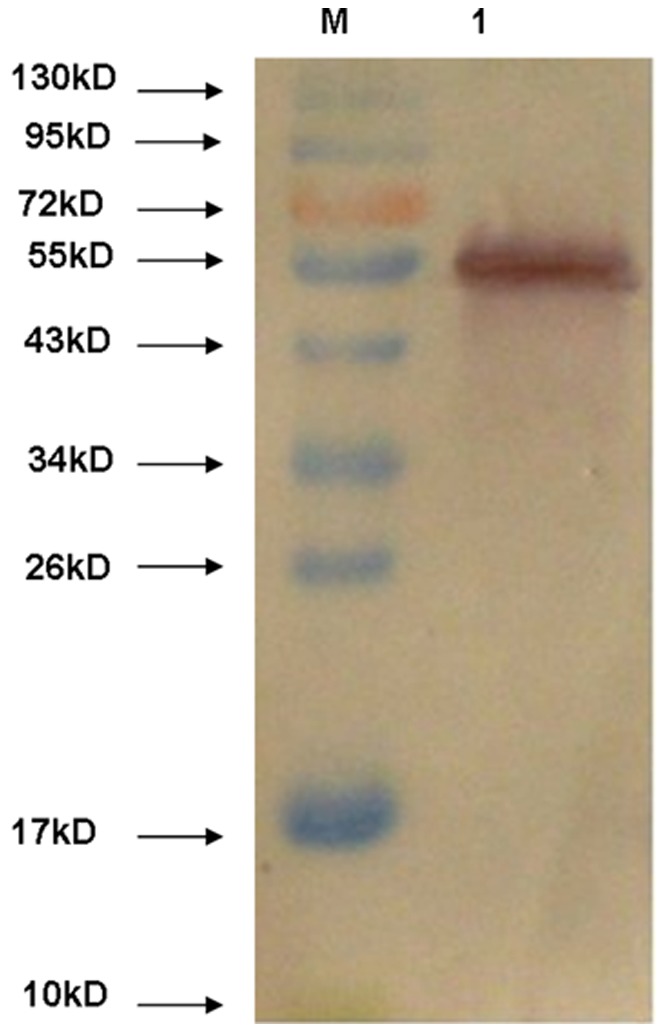
Western blotting analysis of the immunogenicity of rSjMF. Lane M: molecular mass markers; lane 1: purified rSjMF was probed with serum from rabbit immunized against SWAP.

The SjMF gene was cloned into the pET32a(+) expression vector, and the recombinant protein was expressed successfully with an expected size of 62 kDa in *E. coli* BL21 (DE3) cells induced by IPTG. SDS-PAGE showed that the insoluble fractions contained the majority of the recombinant protein, which was mostly soluble by extraction with 8 M urea. rSjMF protein was purified by affinity chromatography using His binding columns under denaturing conditions, and was then refolded by dialysis against PBS containing successive decreasing concentrations of urea. rSjMF was identified further by western blot using serum from rabbits immunized against SWAP (soluble adult worms antigen preparation) and naive rabbit serum. A positive band of 62 kDa was observed when probed with rabbit serum specific to SWAP, but not in the naive rabbit serum, which revealed that rSjMF had good immunogenicity (Fig. 7).

### 6. rSjMF Immunization Elicits Partial Protection against Schistosomal Infection and Reduction in Liver Egg Number

Compared with the adjuvant control group, mice vaccinated with rSjMF had lower worm burdens and liver egg numbers (Table 1). Mice immunized with rSjMF showed 23.21% and 21.82% reductions in worm burden (P<0.05) as well as 28.35% and 50.52% reductions in liver egg numbers (P<0.01) compared to adjuvant control groups in two independent trials.

**Table 1:** Protection induced in mice immunized with rSjMF.

Group	Worm burdenmean ±SD	Percent reduction inworm burden (%)	Eggs/g liver mean ±SD	Percent reduction in liveregg count (%)
Trial 1				
PBS+ISA206	23.83±5.56		65288.62±17063.6	
SjMF-pET32a(+)	18.30±5.35	23.21% *	46779.60±10360.37	8.35%**
Trial 2				
PBS+ISA206	26.86±3.44		106090.4±34737.25	
SjMF-pET32a(+)	21.00±4.27	21.82%*	60916.48±27713.21	42.58%**

Data are expressed as mean±SD and statistically significant compared to control group shown by *P<0.05 or **P<0.01.

### 7. Specific Antibody Responses Induced by rSjMF Immunization

**Figure 8:**
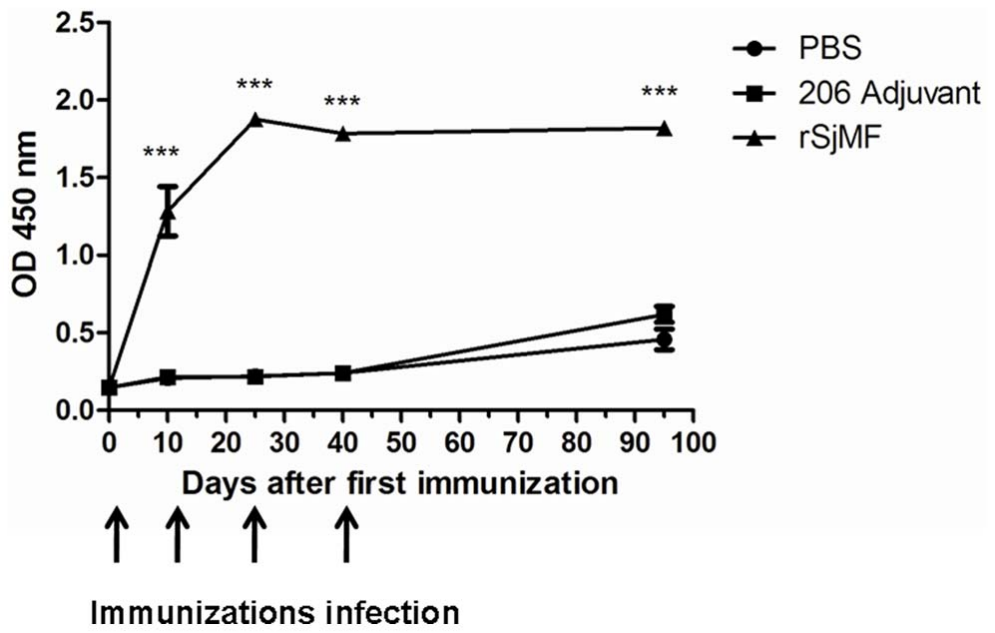
Kinetics of specific anti-rSjMF IgG in mice. Mice were immunized with three doses of a formulation containing rSjMF (20 µg/mouse) or saline in association with ISA 206 adjuvant. Serum samples were obtained from 10 individual mice from each group on days 0,10, 25, 40 and 95 after the first immunization and assayed by ELISA. Arrows indicate the time of each immunization and challenge infection. Results are presented as mean absorbance measured at 450 nm. Significantly increased serum antibody titers compared with the PBS control is denoted by ***P<0.001.

ELISA showed that in the serum of rSjMF-immunized mice, the levels of anti-rSjMF total IgG antibody were significantly higher compared to those in adjuvant control groups after the first immunization, which were maintained until the mice were killed. In the blank and adjuvant control groups, there were no significant differences in specific total IgG levels before or after vaccination (Fig. 8). To evaluate the IgG subtype in the immune response elicited by rSjMF immunization, the levels of IgG1 and IgG2a antibodies specific to rSjMF were also determined by ELISA. No significant changes were observed in the adjuvant control group, and rSjMF immunization induced significant production of specific anti-rSjMF IgG1 and IgG2a antibodies compared to the control group after the first immunization (Table 2).

**Table 2:** Specific IgG1 and IgG2a antibody level in rSjMF-vaccinated or blank control mice.

	IgG1		IgG2a		
Days	ISA 206	rSjMF-adjuvant	ISA 206	rSjMF-adjuvant	IgG1/IgG2a ratio
0	0.083±0.0075	0.078±0.0034	0.073±0.0060	0.068±0.0031	1.15
10	0.096±0.012	0.75±0.17**	0.072±0.014	0.086±0.010	8.72
25	0.081±0.0028	0.99±0.032***	0.070±0.0057	0.44±0.26*	2.25
40	0.11±0.020	0.91±0.046***	0.064±0.0067	0.66±0.25**	1.38

Significantly increased serum antibody titers compared with the ISA 206 adjuvant control is denoted by *P<0.05, **P<0.01 or ***P<0.001.

### 8. rSjMF plus ISA206 Immunization Induces a Mixed Th1/Th2 Response

**Figure 9:**
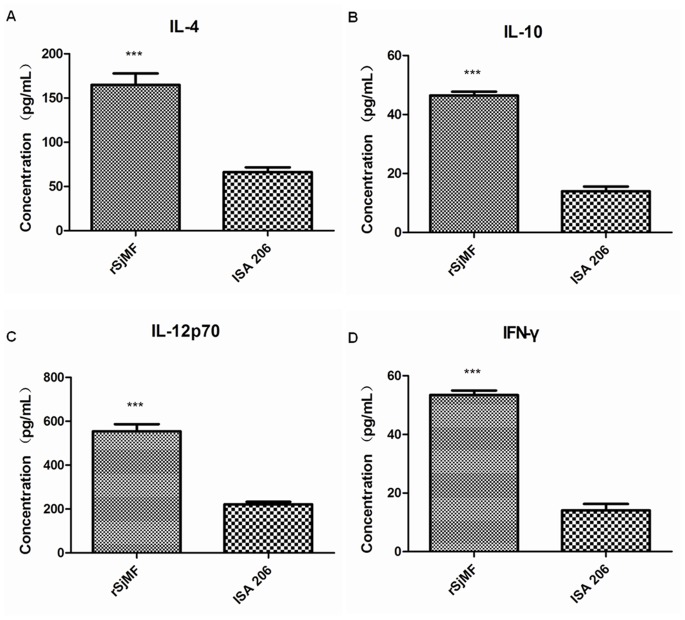
Cytokine profile of mice immunized with rSjMF. Seven days after the last immunization, serum was isolated and assayed for IL-4, IL-10, IL-12 and IFN-γ production in response to rSjMF or ISA 206 adjuvant alone, as a control. Significantly increased serum cytokine concentration compared with the ISA 206 adjuvant control is denoted by **P<0.01 or ***P<0.001.

To determine the cytokine profile produced by vaccination with rSjMF, seru from mice after immunization with rSjMF plus 206 adjuvant or 206 adjuvant in PBS were analyzed by the Luminex 100 multiplex bead-based immunoassay system. Mice in the vaccination group had significantly higher concentrations of IL-4, IL-10, IL-12p70 and IFN-γ compared with the control group (Fig. 9). IL-12p70 and IFN-γ are indicative of a Th1-type immune response, and IL-4 are the characteristic cytokines of a Th2 immune response. These results revealed that rSjMF plus ISA206 immunization induces a mixed Th1/Th2 response.

## Discussion

Schistosomiasis is one of the most important neglected tropical diseases and continues to be a significant public health problem worldwide. The effective control strategy for this zoonosis is to develop vaccines and improve sanitation. The schistosomal tegumental proteins are not just key molecules for worms to survive in their host, but also targets for the host’s immune attack and drug treatment. Previous studies have demonstrated that some membrane proteins successfully induce protective immune response against schistosomal infection, such as Sj23, SmTSP-2 and Sm29 [7], [9], so it is possible to screen for more vaccine candidates or drug targets by further investigation of schistosomal tegumental proteins. Recently, we have isolated 85 tegumental proteins from 42-day adult worms via *S. japonicum* tegument surface protein analysis, including myoferlin, dysferlin and some other important schistosomal proteins, which provides the basis for us to identify more effective vaccine candidates or new drug targets for the control of schistosomiasis (data not published).

In the present study, one of the schistosomal tegumental proteins SjMF was characterized, cloned and expressed, and the potential of rSjMF as a vaccine candidate against schistosomal infection in mice was evaluated.

Bioinformatic analysis revealed that SjMF contained the conserved domain of the ferlin family that is involved in vesicle fusion. Therefore, we propose that myoferlin might be a part of the emergency response that mediates resealing-based fusion events in the tegument and muscle of *S. japonicum*.

Besides, PZQ could disturb the transcription of myoferlin. We treated mice at 35 days post-infection with high- and low-dose PZQ, and compared the transcript level of SjMF in worms in the treated and untreated groups with RT-PCR. SjMF was significantly inhibited at 36 h after treatment with a single dose of 200 mg/kg PZQ, accompanied with unrecovered tegumental damage. However, SjMF was upregulated significantly at 12 and 36 h after treatment with a single dose of 40 mg/kg PZQ, accompanied with recovery of tegumental damage. Moreover, many large vacuoles occurred at 4 h after treatment with 40 mg/kg PZQ, and the vacuoles decreased and turned substantial after 36 h. However, the vacuoles did not change at 36 h after treatment with 200 mg/kg PZQ. In the low-dose PZQ group, the worms were not killed and recovered their normal morphological features. Moreover, Davis et al. have carried out a series of elegant experiments that have shown that myoferlin is associated with the plasma and nuclear membranes [11], [20], suggesting that myoferlin is potentially an essential protein for plasma membrane integrity. Therefore, we speculate that SjMF may participate in maintaining the integrity of plasma membrane of *S. japonicum*.

Sequence analysis displayed that the ORF of SjMF was conserved in all stages of the *S. japonicum* life cycle. And real-time PCR analysis showed that SjMF transcripts were expressed in all stages of *S. japonicum* tested, with a higher expression level in 42-day worms. Additionally, the expression level in the female worms at 42 days was significantly higher than that in their male counterparts. It is well known that the female worms of *S. japonicum* begin to lay eggs at 24 days [21]. During this process, more materials are needed for the development of the ovaries and vitellaria, which are involved in vesicle trafficking and fusion, while, myoferlin has been proposed to facilitate vesicle trafficking and fusion during membrane repair [22], [23], suggesting that SjMF is important for the development of the schistosome.

Immunolocalization studies showed that SjMF was primarily located in the tegument of *S. japonicum*, which is supported by the recent proteomics studies on the tegumental surface protein of *S. japonicum* in our laboratory. Western blotting showed that rSjMF had good immunogenicity, and mice with rSjMF had a partial but significant reduction of 21.8% to 23.21% and 42.58% to 28.35% for mean worm burden and liver egg burden, respectively. Although the number of worms and eggs found between the two independent trials were different (Table 2), the trend for reduction in worm and liver egg burdens in the two trials was similar, suggesting that rSjMF immunization not only reduced worm burden, but also reduced egg number. Eggs produced by female worms are important in disease transmission and are responsible for pathogenesis. Therefore, it is meaningful to develop an antipathological vaccine based on reduced egg burden in the liver.

The humoral immune response to rSjMF was evaluated. Significantly high levels of anti-rSjMF IgG were detected after immunization with rSjMF and maintained until the mice were killed, which may correlate with the protective efficacy induced by rSjMF. IgG isotype analysis showed that rSjMF immunization induced significant production of both IgG1 and IgG2a specific antibodies, and specific IgG1 level remained higher than that of IgG2a. Furthermore, the relative quantities of IgG1 and IgG2a could be regulated by the differential secretion of IL-4 or IFN-γ [24]. IL-4 can stimulate IgG1 production, whereas IFN-γ can suppress IgG1 production and enhance IgG2a production [25]. In this study, the production of Th1 and Th2 cytokines was significantly higher than in the control group, therefore, we conclude that SjMF induced a mixed Th1/Th2 immune response.

Taking all these findings together, we speculate that, just like Sm23, SmTSP and some other tegumental proteins, rSjMF might be a vaccine candidate for schistosomiasis control.

In conclusion, SjMF is a tegumental membrane-anchored protein that may be a potential vaccine candidate for schistosomiasis,. Further investigations are required to elucidate fully the function of this molecule.

## References

[1] Wang L, Utzinger J, Zhou XN (2008) Schistosomiasis control: experiences and lessons from China. Lancet 372(9652): 1793–5. 18930529 10.1016/S0140-6736(08)61358-6 PMC7135384

[2] Gryseels B, Polman K, Clerinx J, Kestens L (2006) Human schistosomiasis. Lancet 368 (9541): 1106–18. 10.1016/S0140-6736(06)69440-3 16997665

[3] van der Werf MJ, de Vlas SJ, Brooker S, Looman CW, Nagelkerke NJ, et al (2003) Quantification of clinical morbidity associated with schistosome infection in sub-Saharan Africa. Acta Trop 86 (2–3): 125–39. 10.1016/s0001-706x(03)00029-9 12745133

[4] Caffrey CR, Secor WE (2011) Schistosomiasis: from drug deployment to drug development. Curr Opin Infect Dis 24 (5): 410–7. 10.1097/QCO.0b013e328349156f 21734570

[5] Carod AF (2012) Cerebral and Spinal Schistosomiasis. Curr Neurol Neurosci Rep 5 (9): 1420–34. 10.1007/s11910-012-0305-4 22903225

[6] Pearce EJ, MacDonald AS (2002) The immunobiology of schistosomiasis. Nat Rev Immunol 2 (7): 499–511. 10.1038/nri843 12094224

[7] Tran MH, Pearson MS, Bethony JM, Smyth DJ, Jones MK, et al (2006) Tetraspanins on the surface of Schistosoma mansoni are protective antigens against schistosomiasis. Nat Med 12 (7): 835–40. 10.1038/nm1430 16783371

[8] Da’Dara AA, Skelly PJ, Wang MM, Harn DA (2001) Immunization with plasmid DNA encoding the integral membrane protein, Sm23, elicits a protective immune response against schistosome infection in mice. Vaccine 20 (3–4): 359–69. 10.1016/s0264-410x(01)00374-7 11672898

[9] Cardoso FC, Macedo GC, Gava E, Kitten GT, Mati VL, et al (2008) Schistosoma mansoni tegument protein Sm29 is able to induce a Th1-type of immune response and protection against parasite infection. PLoS Negl Trop Dis 2 (10): e308. 10.1371/journal.pntd.0000308 PMC2553283 18827884

[10] McManus DP, Loukas A (2008) Current status of vaccines for schistosomiasis. Clin Microbiol Rev 21 (1): 225–42. 10.1128/CMR.00046-07 PMC2223839 18202444

[11] Davis DB, Delmonte AJ, Ly CT, McNally EM (2000) Myoferlin, a candidate gene and potential modifier of muscular dystrophy. Hum Mol Genet 9 (2): 217–26. 10.1093/hmg/9.2.217 10607832

[12] Demonbreun AR, Lapidos KA, Heretis K, Levin S, Dale R, et al (2010) Myoferlin regulation by NFAT in muscle injury, regeneration and repair. J Cell Sci 123 (Pt 14): 2413–22. 10.1242/jcs.065375 PMC2894657 20571050

[13] Doherty KR, Demonbreun AR, Wallace GQ, Cave A, Posey AD, et al (2008) The endocytic recycling protein EHD2 interacts with myoferlin to regulate myoblast fusion. J Biol Chem 283 (29): 20252–60. 10.1074/jbc.M802306200 PMC2459265 18502764

[14] Robinson JM, Ackerman WT, Behrendt NJ, Vandre DD (2009) While dysferlin and myoferlin are coexpressed in the human placenta, only dysferlin expression is responsive to trophoblast fusion in model systems. Biol Reprod 81 (1): 33–9. 10.1095/biolreprod.108.074591 PMC3093985 19228595

[15] Gobert GN, Moertel L, Brindley PJ, McManus DP (2009) Developmental gene expression profiles of the human pathogen Schistosoma japonicum. BMC Genomics 10: 128. 19320991 10.1186/1471-2164-10-128 PMC2670322

[16] Hong Y, Han H, Peng J, Li Y, Shi Y, et al (2010) Schistosoma japonicum: cloning, expression and characterization of a gene encoding the alpha5-subunit of the proteasome. Exp Parasitol 126 (4): 517–25. 10.1016/j.exppara.2010.06.029 20599988

[17] Thomas JM, Zewail AH (2010) 4D electron microscopyimaging in space and time. London: Imperial College Press. 341 p.

[18] Smithers SR, Terry RJ (1965) The infection of laboratory hosts with cercariae of Schistosoma mansoni and the recovery of the adult worms. Parasitology 55 (4): 695–700. 10.1017/s0031182000086248 4957633

[19] Xiao SH, Shen BG, Horner J, Catto BA (1996) Tegument changes of Schistosoma japonicum and Schistosoma mansoni in mice treated with artemether. Zhongguo Yao Li Xue Bao 17 (6): 535–7. 9863150

[20] Davis DB, Doherty KR, Delmonte AJ, McNally EM (2002) Calcium-sensitive phospholipid binding properties of normal and mutant ferlin C2 domains. J Biol Chem 277 (25): 22883–8. 10.1074/jbc.M201858200 11959863

[21] He YX, Yang HZ (1980) Physiological studies on the post-cercarial development of Schistosoma japonicum. Acta Zoologica Sinica 26: 32–39.

[22] Mellgren RL, Zhang W, Miyake K, McNeil PL (2007) Calpain is required for the rapid, calcium-dependent repair of wounded plasma membrane. J Biol Chem 282 (4): 2567–75. 10.1074/jbc.M604560200 17121849

[23] Mellgren RL, Miyake K, Kramerova I, Spencer MJ, Bourg N, et al (2009) Calcium-dependent plasma membrane repair requires m- or mu-calpain, but not calpain-3, the proteasome, or caspases. Biochim Biophys Acta 1793 (12): 1886–93. 10.1016/j.bbamcr.2009.09.013 PMC2787696 19781581

[24] Finkelman FD, Holmes J, Katona IM, Urban JJ, Beckmann MP, et al (1990) Lymphokine control of in vivo immunoglobulin isotype selection. Annu Rev Immunol 8: 303–33. 1693082 10.1146/annurev.iy.08.040190.001511

[25] Coffman RL, Carty J (1986) A T cell activity that enhances polyclonal IgE production and its inhibition by interferon-gamma. J Immunol 136 (3): 949–54. 2934482

